# DASH Diet as a Proposal for Improvement in Cellular Immunity and Its Association with Metabolic Parameters in Persons with Overweight and Obesity

**DOI:** 10.3390/nu13103540

**Published:** 2021-10-09

**Authors:** Carmen Paulina Rodríguez-López, María Cristina González-Torres, Carlos A. Aguilar-Salinas, Oralia Nájera-Medina

**Affiliations:** 1 Departamento de Atención a la Salud, CBS, Universidad Metropolitano México-Xochimilco (UAM-Xochimilco), Mexico City 04960, Mexico; cprl04@xanum.uam.mx; 2 Departamento de Ciencias de la Salud, CBS, Universidad Autónoma Metropolitano-Iztapalapa (UAM-Iztapalapa), Mexico City 09340, Mexico; mcgt@xanum.uam.mx; 3 Departamento de Endocrinología y Metabolismo, Instituto Nacional de Ciencias Médicas y Nutrición Salvador Zubirán (INCMNSZ), Mexico City 14080, Mexico; caguilarsalinas@yahoo.com

**Keywords:** obesity, chronic inflammation, dietary pattern

## Abstract

The development of obesity entails a chronic low-grade inflammatory state with increased pro-inflammatory cells, mainly in visceral adipose tissue (VAT). Additionally, dietary patterns have an influence on the regulation of chronic inflammation. Dietary Approaches to Stop Hypertension (DASH) include foods with an anti-inflammatory profile and that have positive impacts on body composition (BC), suggesting improvements in inflammatory processes. Objective: To analyze the impact of the DASH diet on cellular immunity, anthropometric, biochemical and BC parameters in patients with overweight and obesity, who could present metabolic syndrome. Methodology: Lymphocyte subpopulations, biochemical parameters, anthropometric parameters, and BC before and 8 weeks after intervention with the DASH diet in persons with overweight and obesity were measured. Results: Fifty-nine young adults participated in the study. After the intervention, no significant changes in biochemical parameters were observed, although a significant decrease in nearly all of the anthropometric and BC variables was found: waist circumference (*p* < 0.001), percentage and kilograms of fat (*p* < 0.001 and *p* < 0.025, respectively), VAT (*p* < 0.020), and weight (*p* < 0.001), as well as total lymphocytes and double-positive TCD4+ cells. A relation between changes in leukocyte subpopulations (monocytes, natural killer, helper and cytotoxic lymphocytes, and naive TCD4+ cells) and metabolic improvements (glucose, triglycerides, total cholesterol and LDL-c) was also found. Conclusions: The DASH diet promotes positive changes in lymphocyte subpopulations, anthropometric parameters and BC in persons with overweight and obesity. Future studies should elucidate the cellular and molecular mechanisms through which the DASH diet produces inmunometabolic improvement.

## 1. Introduction

Overweight (OW) and obesity (Ob) are considered a global public health problem as they increase the risk of developing metabolic syndrome (MS), type 2 diabetes, and cardiovascular diseases, among other alterations [1]. It has been observed that the development of obesity entails a chronic low-grade inflammatory state, which involves an increase in pro-inflammatory immune cells (neutrophils, lymphocytes TCD8, NK, B and macrophages type 1) in relation to anti-inflammatory cells (regulatory T lymphocytes and macrophages type 2) within adipose tissue (AT), mainly in visceral adipose tissue (VAT) [2,3,4,5,6,7].

Chronic inflammation has been demonstrated to have systemic consequences as it entails the production and secretion of pro-inflammatory cytokines and chemokines, which are, in turn, a by-product of the interaction between cells in the VAT. Cytokines and chemokines have endocrine effects that contribute to systemic insulin resistance when impacting on other organs (liver, pancreas and skeletal muscle) [4,8,9,10], thus preceding cardiometabolic comorbidities related to obesity [2].

Effective actions are required to prevent the increase in obesity prevalence and to treat persons already affected by it. Lifestyle-based weight-loss programs, which include diets with caloric restriction and increased physical activity, are the most commonly employed methods [11]. Additionally, it has been demonstrated that the quality of feeding affects the inflammatory response of the immune system. A diet rich in fat, refined carbohydrates and protein is commonly associated with high levels of inflammation, while a diet rich in fruits, vegetables and fish is associated with low levels of inflammation or even anti-inflammatory patterns [12,13,14]. Therefore, dietary patterns exert an influence on the regulation of chronic inflammation, establishing diet as a factor that impacts inflammation in patients with obesity [11,15].

The Dietary Approaches to Stop Hypertension (DASH) diet is a dietary plan that includes foods rich in magnesium, potassium and calcium to stop and prevent arterial hypertension [16,17,18]. In addition, the DASH diet includes whole grains, fruits, vegetables, legumes, low-fat dairy products, poultry and fish, together with reduced amounts of saturated fat, cholesterol, red meat and refined grains and sweets [16,19,20,21,22]. Such foods included in the DASH diet are associated with a diet with an anti-inflammatory profile.

Some studies have implemented a DASH diet in persons with metabolic alterations, as in the case of MS [23]. The American Heart Association (AHA) even recommends this type of diet to prevent cardiovascular diseases [16,21,24]. Weight reduction has also been reported to be more significant in participants with OW and Ob who followed a low-calorie DASH diet than in those who followed other low-energy diets [25]. Therefore, the objective of this study was to analyze the impact of a DASH diet on cellular immunity, anthropometric, biochemical and body composition (BC) parameters in patients with OW and Ob, who could present MS.

## 2. Materials and Methods

### 2.1. Study Design

A longitudinal and clinical trial study was performed with young adults, in Mexico City, Mexico. At baseline and after the intervention (8 weeks) with a DASH diet, analysis of the percentage of peripheral leukocyte subpopulations, anthropometric measurements, body composition (bioelectrical impedance analysis and dual-energy X-ray absorptiometry) and biochemical tests were conducted.

### 2.2. Eligibility Criteria

Inclusion criteria: male and female adults aged 18 to 40, both genders. Exclusion criteria: individuals with any type of infection, pregnant, with TDM1 or T2DM, with autoimmune, hepatic, renal, endocrine or heart disease, cancer and those on medication. Elimination criteria: patients who did not sign the informed consent form, who decided to withdraw from the study, and who did not attend or were sick at their second evaluation. Participants provided written informed consent before participation. The study protocol was approved by the Local Ethics Committee of the Metropolitan Autonomous University-Xochimilco, in Session 13/16, held on 1 December 2016, Mexico City, Mexico.

### 2.3. Clinical Measurements

#### 2.3.1. Anthropometric

The assessed measurements included weight, height, and waist circumference (WC), following the standardized protocol of the International Society for the Advancement of Kinanthropometry (ISAK) [26]. A SECA 213 stadiometer set at a precision of 0.1 cm was used for the measurement of height. WC was measured utilizing a flexible and inextensible measuring tape (Lufkin); cutoffs are indicated by the Mexican Ministry of Health considering comorbidity risk factors (T2DM, hypertension and cardiovascular disease): men ≥90 cm, women ≥80 cm [27]. Body mass index (BMI) was calculated as body mass divided by height squared (kg/m^2^). Participants were classified according to the World Health Organization [28] criteria for adults: normal weight: 18.5–24.99 kg/m^2^, overweight: 25–29.99 kg/m^2^, and obesity: ≥30 kg/m^2^.

#### 2.3.2. Body Composition

Body composition was assessed using the InBody720 multi-frequency impedance body composition analyzer with eight-point tactile electrodes equipment. Individuals with ≥100 cm^2^ of VAT were diagnosed as having visceral obesity (VAT increased), while those with <100 cm^2^ were diagnosed as having normal VAT [29]. Weight was also measured with the equipment previously mentioned; all participants were assessed wearing light underwear. Percentage of subcutaneous fat (SF), skeletal muscle mass (SMM), and fat mass (FM) was measured using dual-energy X-ray absorptiometry (DXA; Hologic Discovery) and analyzed employing the Hologic APEX software, version 3.3.0.1.

#### 2.3.3. Biochemical Tests and Determination of Metabolic Syndrome

Blood samples (5 mL) were collected in the morning and after a 12 h overnight fast. Triglycerides (TG), high-density cholesterol (HDL-c), fasting glycemia (Glu), and total cholesterol (Total Cho) were measure with an automated IKEM clinical chemistry analyzer. Low-density cholesterol (LDL-c) was calculated with the Friedewald formula.

Systolic and diastolic blood pressure were measured twice in each patient using a sphygmomanometer with the patient in a semi-reclined position after a rest period, according to the guidelines of the Mexican Official Norm [30] for the Prevention, Treatment, and Control of Hypertension.

The presence of metabolic syndrome (MS) was determined according to the definition of the Cholesterol Education National Program (ATP III), modified for Hispanic people. These guidelines suggest that MS should be considered when there is an association of at least three of the conditions included in Table 1.

#### 2.3.4. Leukocyte Measurement

A second venous blood sample (5 mL) was obtained from patients in order to identify leukocyte subpopulations. Leukocytes were stained using anti-human monoclonal antibodies conjugated to fluorescein (FITC), phycoerythrin (PE), the peridinin-chlorophylla protein (PerCP), and/or allophycocyanin (APC). Combinations of conjugated monoclonal antibodies were used as follows: FITC-anti-CD45/Pe-anti-CD14; FITC-anti-CD3/PE-anti-CD16+CD56/PerCP-anti-CD19; FITC-anti-CD4/PE-anti-CD62/PerCP-anti-CD3; FITC-anti-CD8/PE-anti-CD28/PerCP-anti-CD3; and FITC-anti-CD45RA/PE-anti-CD45RO/PerCP-anti-CD4/APC-anti-CD3. Percentages of total lymphocytes, monocytes and granulocytes were obtained through the first combination; TCD3+ lymphocytes, NK, and B with the second combination; TCD4+ lymphocytes, helper cells (TCD4+CD62-) and TCD3+ were obtained through the third one; TCD8+ lymphocytes, cytotoxic cells (TCD8+CD28-) and TCD3+ through the fourth one; and memory (CD45RO+) and naive (CD45RA+) cells of TCD3+ and TCD4+ through the last one. Cells were incubated with antibodies under conditions of darkness for 20 min. Then, 3 mL of lysis solution was added and incubated for 10 min. After washing, cells were fixed with 1% *p*-formaldehyde and stored under conditions of darkness until analyzed through flow cytometry (within the first 24 h) [32,33].

Sample analysis was performed using a FACSCanto II Cytometer with FACSDiva software (BD Biosciences, San Jose, CA, USA); 10,000 cells were counted in each sample. Two fluorescence dual-axis graphs were constructed.

### 2.4. Intervention

The dietary plan was based on the DASH diet, utilizing its percentages of macronutrient distributions as follows: 55% carbohydrates; 27% lipids, and 18% proteins. This diet also takes into consideration the specific parameters of sodium, potassium, calcium, and magnesium; however, in the present work, these were not considered quantitatively: patients were only advised to prefer foods that contained these micronutrients, and they were offered a list of these nutriments.

The calculation of total energy had, as its objective, a weight reduction of 500 g per week. To achieve this objective, the total energy requirement was calculated using the Food and Agricultural Organization/World Health Organization (FAO/WHO) equation of 2004, to which kilocalories (Kcal) obtained from a Food Consumption Frequency (FCC) questionnaire of each patient, designed for the Mexican population, were added [34,35]. The average was calculated and 500 Kcal were subtracted from this number [36].

A diet plan, personalized to the highest extent possible, was carried out in order to achieve the highest adherence of the patient. Follow-up through the patient’s subsequent appointments was carried out every 2–3 weeks, according to the performance of each patient.

For the elaboration of the dietary plan, work was conducted based on the Sistema Mexicano de Alimentos Equivalentes (SMAE, Mexican System of Food Equivalents) [37]. The number of grams of each macronutrient was transformed into food rations distributed among each of the food groups. Regarding calories specific to each individual, its distribution among different food groups was adapted according to the following general recommendation (2000 kcal/day diet): 6–8 portions of whole grains, 4–5 fruits and vegetables, about 7 carbohydrates, 2 or less lean meat products and 2–3 portions of skimmed dairy products. Dehydrated fruits, seeds, and legumes were to be ingested at least 4 times a week [16,38]. The sodium content of the DASH diet was designed to be less than 2400 mg/day [39]. The distribution of the rations had, as its final purpose, the achievement of distributing these in three main meals (breakfast, lunch, and dinner) and in two snacks (morning and evening).

To achieve adherence, participants were oriented through the benefits of a healthy diet (for example, the incorporation of fruits, vegetables, whole grains, etc.) and food portions. They were provided with practical guidelines that aided them in selecting foods that were appropriate for their diet. Participants were also supported in elaborating a menu based on their dietary habits and in compliance with the DASH diet guidelines.

### 2.5. Statistical Analysis

The Shapiro–Wilk test was utilized to determine whether the variables presented had a Gaussian distribution. Logarithmic transformation was performed to approximate normality in variables without Gaussian distribution. The results are presented as mean ± standard deviation (SD) and the median, with the interquartile range (IQR, 25–75). Comparisons between baseline conditions and those present after the intervention were conducted with a Student t test for paired samples. One-way ANOVA was applied to determine differences among >2 groups as well as Bonferroni post hoc tests to find out among which groups differences were present. Data were adjusted by sex and age. A *p* value of <0.05 was considered significant, using the SPSS ver. 21 statistical software program.

## 3. Results

**Figure 1:**
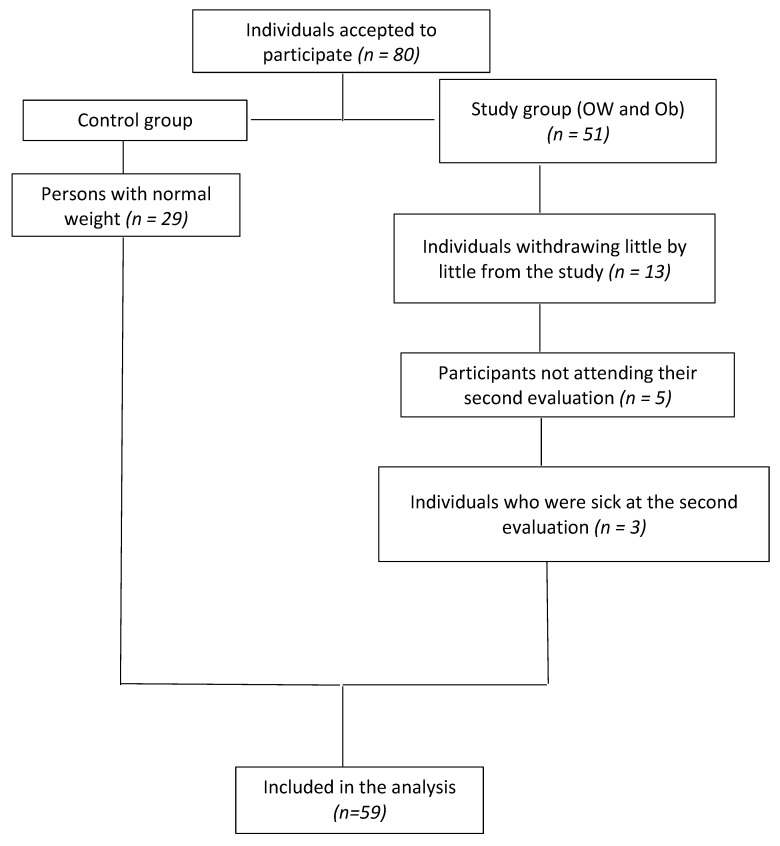
Distribution of the population along the study period.

In the present study, 80 persons were initially incorporated; however, due to individual circumstances, 13 withdrew, 5 did not attend the second evaluation, and 3 more were sick at their second evaluation; therefore, these participants were eliminated from the study (Figure 1).

In the analysis, 59 subjects were included and distributed in two groups: the control group and the study group. The former included 29 participants with normal body mass index (BMI) and VAT, without MS (individuals who did not follow a food plan), and paired by sex and age with the study group. The latter included 30 persons with OW and Ob, who adhered to a dietary plan for weight reduction (Figure 1). The mean age of all participants was 25.9 ± 6.5 years. Ninety percent of participants were female.

### 3.1. Baseline Characteristics

Of the total number of subjects included in the study (*n* = 59), 49% (*n* = 29) had a normal BMI, 24% (*n* = 14) had OW, and 27% (*n* = 16) had Ob. On the other hand, a prevalence of only 13% (*n* = 4) MS was found in persons with OW and Ob. Likewise, it was observed that persons with Ob and MS in their majority presented increased VAT.

### 3.2. Laboratory Analysis, Anthropometric Parameters, and Body Composition

When biochemical variables were analyzed, it was observed that persons with Ob presented higher glucose values than individuals with OW. In addition, in persons with OW and Ob, high-density lipoprotein-cholesterol (HDL-c) was found to be low, while diastolic and systolic arterial pressure were high. Compared with normal weight participants, all these differences were statistically significant (Table 2).

Regarding anthropometric and BC measurements, persons with normal weight exhibited the lowest values in waist circumference (WC), subcutaneous adipose tissue (ST), body fat mass (BFM), and VAT, in comparison with persons with OW and Ob. All these differences were significant (Table 2).

### 3.3. Leukocytes Subpopulation

With respect to lymphocyte subpopulations, it was found that monocytes and naïve TCD3+ (TCD3+CD45RA+) cells decreased as BMI increased; therefore, persons with Ob had the lowest values, while persons with normal weight, the highest. On the other hand, the percentage of NK cells was lower in persons with OW. In terms of TCD8+ cells, it was found that persons with Ob had the lowest values in comparison with persons with OW. The lowest percentages of naïve TCD3+ and TCD4+ (CD45RA+) cells and the highest percentages of memory cells (TCD3+CD45RO+ and TCD4+CD45RO+) were observed in individuals with OW and Ob. All these differences were significant (Table 3).

### 3.4. Implementation of a Dietary Plan for Metabolic and Immunological Improvement

**Figure 2:**
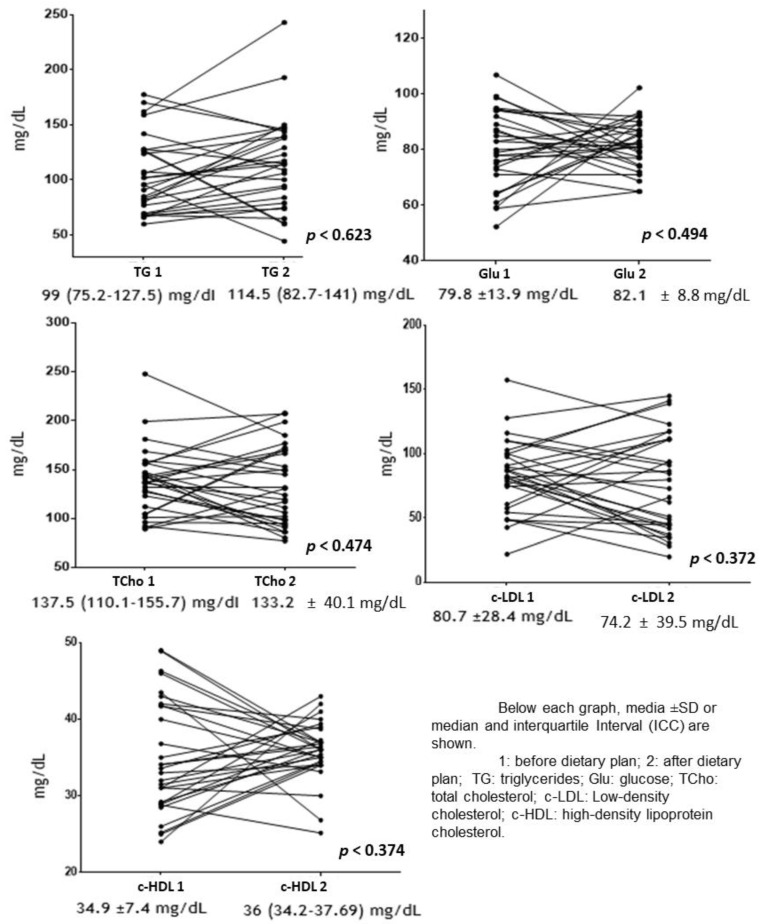
Changes in biochemical indicators before and after dietary plan.

In Figure 2, changes in biochemical indicators of participants, before and after implementation of a dietary plan, are presented, without these being statistically significant. However, when biochemical variables were analyzed in each participant with OW and Ob, it was observed that triglycerides (TG) decreased in 33% of participants by 48.6 ± 15%, glucose in 50% by 11.5 ± 8.6%, total cholesterol in 60% of individuals by 30.5 ± 18.8%, low-density lipoprotein-cholesterol (LDL-c) in 60% by 34.6 ± 17.3%, and HDL-c increased in 56% of individuals by 6.9 ± 4.4% (data not shown). These results were not observed when analyzing these variables by group.

**Figure 3:**
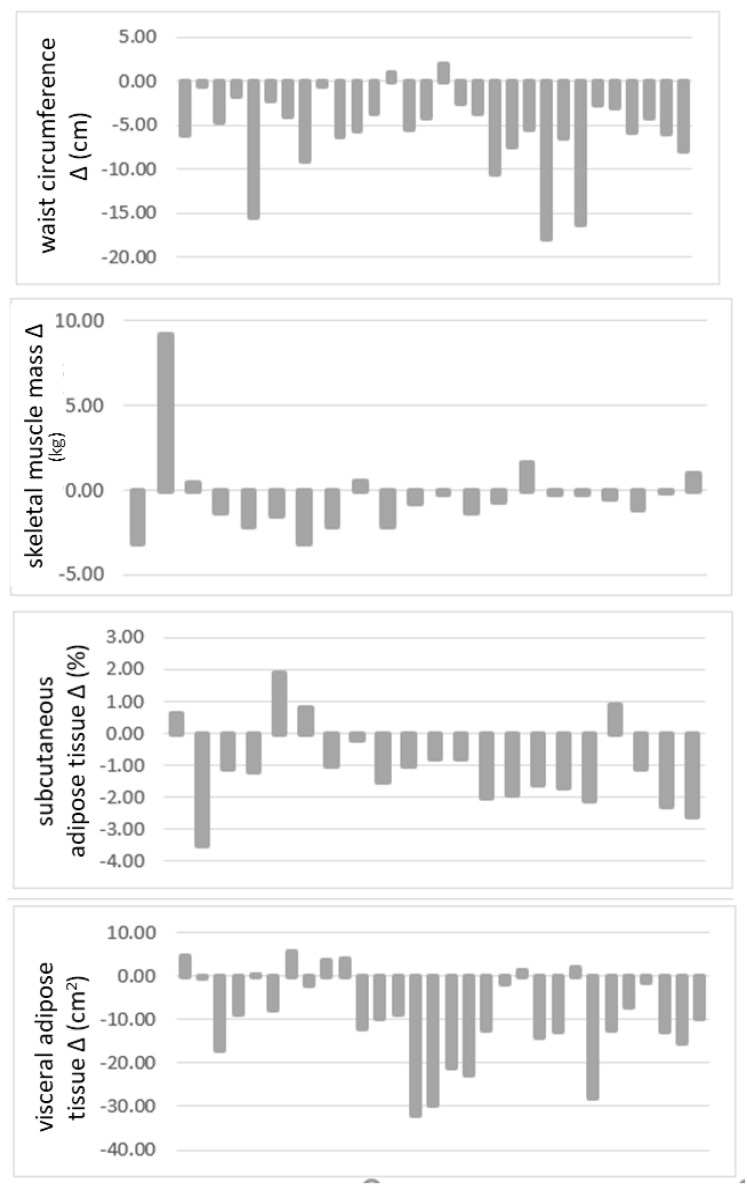
Changes in anthropometric and body composition measurements after the intervention.

In relation to anthropometric and BC measurements after intervention in participants with OW and Ob, it was found that nearly all variables statistically diminished as follows: WC (*p* < 0.001), ST (*p* < 0.001), BFM (*p* < 0.025), VAT (*p* < 0.020), and weight (*p* < 0.001); only the kg of the SMM was maintained (Table 2). In Figure 3, change (Δ) in those previously mentioned variables can be observed in each participant after implementation of the dietary plan.

After analyzing individual results, an improvement in BMI was observed: four persons with Ob became OW and two persons with OW became normal weight. In addition, two persons with visceral obesity became normal (data not shown).

### 3.5. Leukocyte Subpopulations after Intervention

After dietary plan, it was observed in all participants that total lymphocytes and double-positive TCD4+ (TCD4+CD45RA+CD45RO+) cells diminished, while TCD3+ lymphocytes increased. All these changes were statistically significant. The remaining subpopulations presented no changes (data not shown).

On performing the analysis according to BMI, it was found that in persons with OW, total lymphocytes diminished and granulocytes increased after intervention. These differences were statistically significant. Double-positive TCD4+ (TCD4+CD45RA+CD45RO+) presented a tendency to diminish in persons with OW; however, where these indeed diminished significantly was in persons with Ob. In addition, a significant increase in TCD3+ and TCD4+CD45RO+ (memory) lymphocytes and B lymphocytes’ tendency to diminish were found in persons with Ob (Table 3).

### 3.6. Partial Correlations and Linear Regressions after Intervention

Partial correlations were obtained through the relation between changes (Δ) observed in leukocyte cells; upon performing the analysis according to BMI, it was found that in persons with OW, total lymphocytes diminished and granulocytes increased after intervention. These differences were statistically significant. Double-positive TCD4+ (TCD4+CD45RA+CD45RO+) presented a tendency to diminish in persons with OW; however, where these indeed diminished significantly was in persons with Ob. In addition, a significant increase in TCD3+ and TCD4+CD45RO+ (memory) lymphocytes and B lymphocytes’ tendency to diminish were found in persons with Ob (Table 3).

There were Δ in anthropometric, biochemical, and body composition variables after intervention. The analysis was adjusted by sex, age, and BMI. It was found that Δ at peripheral level of monocytes correlated negatively with Δ in DBP but positively with Δ in ST, just as Δ in NK lymphocytes correlated positively with Δ in TG. A negative correlation was found between Δ in TCD4+ helper lymphocytes (TCD4+CD62-, effector) and TCD4+ lymphocyte memory cells (TCD4+CD45RO+) and Δ in WC and glucose, respectively. A positive correlation was also observed between Δ in TCD8+ cytotoxic lymphocytes (TCD8+CD28-, effectors) and Δ in ST, and between Δ in TCD4+ virgin lymphocytes (TCD4+CD45RA+) and Δ in TCho, c-LDL, and WC. All these correlations were significant (Table 4).

**Figure 4:**
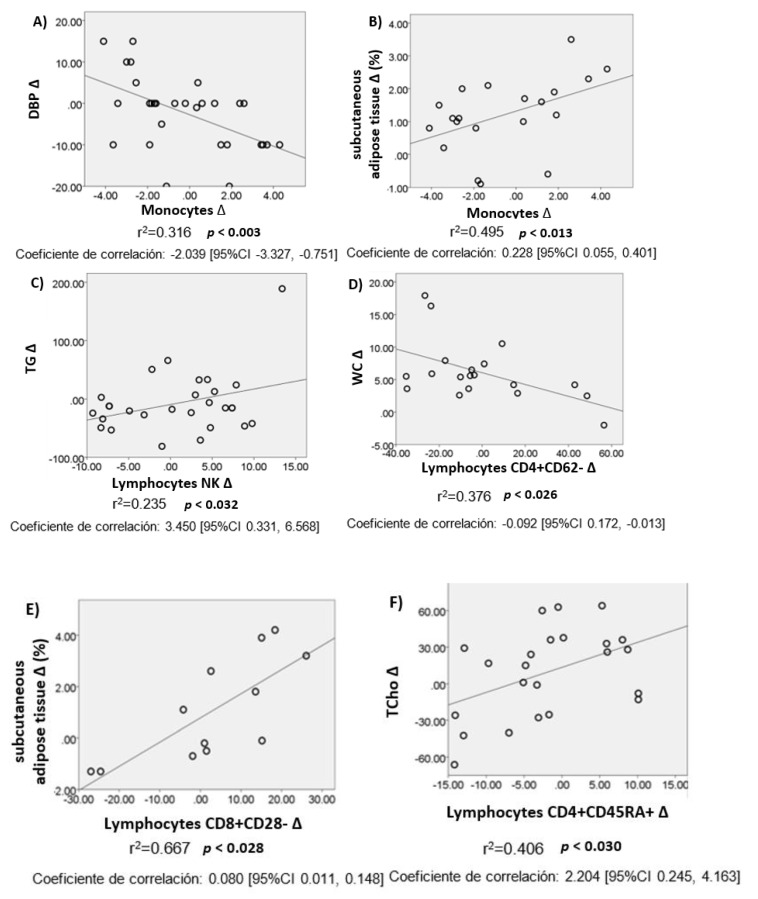

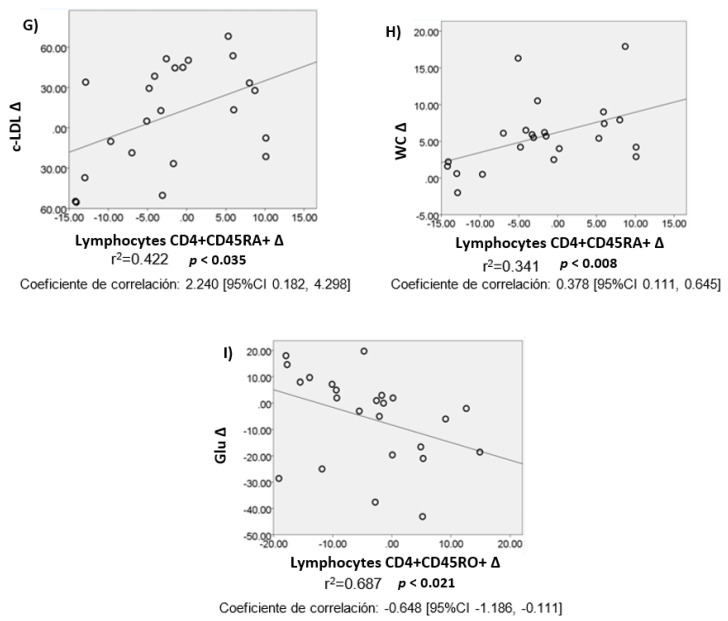
Linear regression between delta in leucocyte cells and delta in anthropometric, biochemical and body composition variables. (**A**) Lineal regression between DBP Δ vs monocytes Δ; (**B**) Lineal regression between ST Δ vs monocytes Δ; (**C**) Lineal regression between TG Δ vs lymphocytes Δ; (**D**) Lineal regression between WC Δ vs Lymphocytes CD4+CD62- Δ; (**E**) Lineal regression between TS Δ vs Lymphocytes CD8+CD28- Δ; (**F**) Lineal regression between TCho Δ vs Lymphocytes CD4+CD45RA+ Δ; (**G**) Lineal regression between c-LDL Δ vs Lymphocytes CD4+CD45RA+ Δ; (**H**) Lineal regression between WC Δ vs Lymphocytes CD4+CD45RA+ Δ; (**I**) Lineal regression between Glu Δ vs Lymphocytes CD4+CD45RO+ Δ. DBP: diastolic blood pressure; TG: triglycerides; WC: waist circumference; TCho: total cholesterol; c-LDL: Low-density cholesterol; Glu: glucose; ST: subcutaneous adipose tissue. *p* value adjusted by sex, age and BMI (*p* < 0.05).

Based on the results above, we decided to carry out a linear regression adjusted by sex, age, and BMI, between Δ in leukocyte subpopulations that correlated statistically and Δ in anthropometric, biochemical, and BC variables. A positive correlation was found between Δ in monocytes and Δ in ST, where a 1% decrease in this tissue is associated with a 1.5% (range, 0.3–2.6) decrease in monocytes (Figure 4).

Δ in TCD8+CD28- lymphocytes also correlated positively with Δ in ST, where a 1% decrease in this tissue was associated with a 6.5% (range, 1–12) decrease in effector lymphocytes (TCD8+CD28-). On the other hand, Δ in TCD4+CD62- effector lymphocytes correlated negatively with Δ in WC, where a 1 cm decrease in WC was associated with a 3% (range, 6–0.4) decrease in TCD4+CD62- lymphocytes. All these data were statistically significant. In Figure 4, all linear regressions with their squared r can be observed.

## 4. Discussion

A poor-quality diet contributes to health adversities, among them overweight and obesity, as well as the comorbidities they entail. Over the last decades years, the population has consumed “unhealthy diets”, characterized by a low intake of fruits, vegetables, seeds, and fish, and a high intake of red meat, processed foods, saturated fats, and sugars in all presentations, which has been associated with an increase in the prevalence of OW and Ob, as well as with deaths caused by heart diseases, heart attacks, and type 2 diabetes mellitus (DM2) [40].

This study focused on observing the impact of the DASH diet on leukocyte subpopulations, and biochemical, anthropometric, and body composition parameters, in persons with OW and Ob.

After 8 weeks under this dietary plan, biochemical variables revealed no significant changes either in the general population or in comparison with BMI. However, when the analysis of each participant was conducted, it was found that TG, glucose, total cholesterol, and LDL-c lowered their blood levels by 30 ± 15%, and HDL-c increased by 6.9 ± 4.4% (in approximately 52% of participants). These data show similarities with those reported by a study in adults with OW and Ob, who adhered to a DASH diet for 3 weeks. These individuals experienced a decrease in total cholesterol, LDL-c, and HDL-c [41]. Similarly, in a longitudinal study carried out in middle-aged women, adherence to the DASH diet was associated with a low risk of cardiovascular disease and heart attacks [42]. In addition, a study in adults with OW and Ob (*n* = 3218, age 39.2 ± 9.5) found that greater adherence to the DASH-style dietary pattern was related to better metabolic profiles [43].

Regarding anthropometric and body composition measurements, we found significant changes in nearly all variables (weight, WC, ST, BFM and VAT). In addition, as previously mentioned, six persons decreased their BMI and two persons decreased their VAT to normality. These data coincide with a study conducted in women (30–40 years, *n* = 60) with OW and OB and polycystic ovary syndrome, where participants lost a significant amount of weight, BMI and body fat [44]. Along these lines, another study in individuals with OW and Ob (age 61.34 ± 7.9, *n* = 131) and primary arterial hypertension found that weight, BMI, WC, and percentage and kilograms of subcutaneous adipose tissue decreased after a 3-month intervention with the DASH diet, although this group had older participants and a longer intervention period than ours [45].

The nutritional intervention carried out in the present study, with a decreased energy intake, was conducted without any additional physical activity in order to assess solely the role of a well-balanced diet in persons with OW and Ob. Important changes in body composition were found, above all in weight and fat loss and mainly in visceral fat (considering that visceral fat is metabolically more active in comorbidities associated with obesity). Therefore, the present results reaffirm the importance of a personalized diet for achieving positive changes in body composition and mainly for VAT loss, which maintains a close relation with the presence of immunological alterations that have repercussions on metabolism [33].

It has been studied how intake of specific nutrients has changed inflammatory parameters in blood and provided a quantitative estimate of the inflammatory tendency of an individual’s diet [11]. In a similar way, feeding affects the nutritional status of individuals, which, in turn, affects the inflammatory response of the immune system. Therefore, malnutrition leads to immunosuppression due to an increased susceptibility to infection, while over-nutrition leads to immunoactivation due to an increased susceptibility to inflammatory conditions [46]. On such basis, we decided to observe the impact of a DASH diet on different leukocyte subpopulations.

In this study, once immune cells were analyzed according to BMI, it was found that total lymphocytes decreased and granulocytes increased only in persons with OW, while TCD3+ lymphocytes and TCD4+ (TCD4+CD45RO+) memory cells increased and double-positive cells TCD4+ (TCD4+CD45RA+CD45RO+) decreased in persons with obesity, thus finding leukocyte mobilization at the differential peripheral level according to BMI.

It was also found that, prior to intervention, participants had increased TCD3+ and TCD4+ (CD45RO+) memory cells. This cell type has been related to a low intensity chronic inflammatory process in persons with obesity [33]; thus, it was expected that these cells would decrease; however, they remained unchanged. Memory T cells are produced through the stimuli of antigens, which, in persons with Ob, are due to the presence of free fatty acids, driving the rise in long-lasting cell population [47].

As above, it has been studied that in persons with OW and Ob, the latter present an imbalance between memory cells (increased) and naïve cells (decreased), which has been associated with premature immunosenescence. Such imbalance and its effect on the immune system is similar to that in older adults [33,48], which limits the host’s ability to mount responses to new antigenic challenge [49]. Another finding of our study with young adults was a moderate increase in naïve cells in persons with OW, perhaps indicating a better immunological balance, as these cells are important to respond to new agents and produce a response toward their elimination [50].

To our best knowledge, there are few studies on the immunological implications of feeding in general in persons with excess weight. A study carried out in 1500 adults (aged 50–69) with OW and Ob found an association between feeding quality (based on the scoring of the DASH diet) and cardiometabolic health markers (a leukocyte count among them). In such study, an inverse association was found between the quality of the DASH diet and the leukocyte count; that is, the higher the score on the DASH diet, the lower the leukocyte count [40], which could be related to the decrease in total lymphocytes found in the present study in individuals with OW—in this case, young adults. Another study reported that a predominance of foods high in cholesterol and saturated fats and relatively low amounts of fruits and vegetables increased inflammation in participants, evidenced by the increase in blood levels of IL-6, homocysteine, and the leukocyte count [13].

It is known that the incorporation of more vegetables, fruits, fish, omega 3 polyunsaturated fats, fiber, and whole grains in the diet (for example, the Mediterranean diet, the macrobiotic diet, etc.) drives the anti-inflammatory potential in persons for whom these foods constitute their habitual diet [12]. These characteristics are shared by the DASH diet, and the changes found in lymphocyte subpopulations could be related to the decrease in the pro-inflammatory process present in this type of person.

It is noteworthy that a 1% decrease in adipose tissue (AT) is associated with a decrease of about 1.5% in monocytes and of 6.5% in TCD8+CD28- lymphocytes, while a 1 cm decrease in WC is associated with a 3% (range, 6–0.4) decrease in TCD4+CD62- lymphocytes (both phenotypes are effector cells). These changes express the impact of diet on body composition and the inflammatory process present in persons with OW and Ob, given that monocytes as well as effector cells have been observed in diverse studies to directly correlate with the degree of metabolic dysfunction in patients with obesity, as these cells provide a link between inflammation in VAT (as well as systemic inflammation) and insulin resistance (IR) through the activation of distinct pro-inflammatory signaling pathways [51,52].

It has been reported that diet plays a central role in the regulation of chronic inflammation [12,15]; however, these are relatively new works, and little has been studied on the pro-inflammatory effect that these interventions have on persons with overweight and obesity. In addition, it has been shown that dietary components have a much broader safety margin than habitual intake of anti-inflammatory pharmaceuticals [12]. Therefore, it is important to continue to work on dietary habits in persons with OW and Ob, as there is evidence, reported by other investigations and supported by the present one, of a positive effect of healthy diets on inflammation variables, as well as on anthropometric, body composition, and biochemical variables.

## 5. Conclusions

It was found that the DASH diet gives rice to positive changes in peripheral immune cells, anthropometric parameters and body composition. In addition, the study found that total lymphocytes and double-positive (TCD4+CD45RO+CD45RA+) cells decreased and TCD3+ lymphocytes increased after intervention. Changes in subpopulations (monocytes, NK lymphocytes, helper, cytotoxic, and naive cells) were related with improvements in glucose, total cholesterol, LDL-c, TG, WC, and ST. These findings could be related with immunological improvement in these patients.

The present study confirms that the DASH diet is a good proposal for nutritious intervention in the treatment of obesity as it improves the chronic inflammatory process, preventing metabolic alterations that could occur in these patients.

## Figures and Tables

**Table 1:** ATP III criteria modified for the Hispanic population.

Criteria	Values	
Triglycerides	≥150 mg/dL	
HDL-c	<40 mg/dL	
Blood pressure	≥130/85 mmHg or previous diagnosis	
Fasting glucose	≥100 mg/dL	
Waist circumference	Women	Men
	≥80 cm	≥90 cm

HDL-c: high-density cholesterol. Adapted from: [31].

**Table 2:** Biochemical indicators, anthropometric measurements and body composition according to BMI, before and after 2 months of the dietary plan.

Variable (*n* = 59)	Normal (*n* = 29)	Overweight (*n* = 14)			Obesity (*n* = 16)			*p* (ANOVA)	
		B	A	*p* ^#^	B	A	*p* ^#^	*p*	*p* (post hoc) *^,&^
TG (mg/dL)	90.7 ± 38.1	101.5 (69.2–131.5)	112 (89.4–138.2)	0.867	99 (77.7–126.7)	115.6 (77.1–145.5)	0.280	0.200	
c-HDL (mg/dL)	50.1 ± 11.6	33.9 ± 7.4 *	36.9 (34.4–39.5)	0.327	35.9 ± 7.5 *	35.5 (34–36.8)	0.824	0.000	0.000
Glu (mg/dL)	78.9 ± 8.3	73.3 ± 13	82.1 ± 10.3	0.107	85.5 ± 12.3 ^&^	82.1 ± 7.6	0.370	0.011	0.009
c-LDL (mg/dL)	70.4 ± 30	70 ± 16.1	72.4 ± 41.3	0.826	90.1 ± 33.8	75.8 ± 37.7	0.154	0.070	
TCho (mg/dL)	138.7 ± 34.4	134 (104.4–143.9)	131.4 ± 41.3	0.439	143 (124–166.1)	134.7 ± 40.3	0.111	0.274	
SBP (mmHg)	101.7 ± 11	105 (100–112.5)	100 (100–116.2)	0.919	110 (110–120) *	110 (100–120)	0.070	0.003	0.002
DBP (mmHg)	69.5 ± 7	70 (67.5-72.5)	70 (70–76.2)	0.328	70 (70–80) *	80 (70-80)	0.855	0.010	0.008
Weight (kg)	55.5 ± 8.3	66.8 ± 7.2 *	64.8 ± 7.2 ^#^	0.001	85.2 ± 10 *^,&^	82.4 ±9.4 ^#^	0.001	0.000	0.000
BMI (kg/m^2^)	21.3 ± 2.1	26.8 (26.4–27.3) *	25.7 (25.1–26.7) ^#^	0.001	31.9 (30.6–35.9) *^,&^	31.7 (29.7–34.7)	0.003	0.000	0.000
WC (cm)	78.4 ± 9.3	89.5 ± 8 *	84.1 ± 6.3 ^#^	0.000	102.4 ± 8.7 *^,&^	96.8 ± 10.3 ^#^	0.001	0.000	0.000
SMM (kg)	31.5 ± 6.2	35.4 ± 7.8	35.6 ± 8.1	0.874	41.1 ± 7 *	40.3 ± 6.9	0.083	0.000	0.000
ST (%)	32.3 ± 5.2	40.7 ± 5.5 *	39.6 ± 5.4 ^#^	0.038	46.1 ± 5.1 *^,&^	45.1 ± 5.1 ^#^	0.021	0.000	0.000
BFM (kg)	16.4 ± 3.7	26.3 ± 3.6 *	25.2 ± 4.1 ^#^	0.025	38.6 ± 5.8 *^,&^	36.3 ± 5.7 ^#^	0.001	0.000	0.000
VAT (cm^2^)	61 ± 21.4	111.3 ± 22.3 *	106.3 ± 24.6 ^#^	0.020	174 ± 38.6 *^,&^	161 ± 34.6 ^#^	0.000	0.000	0.000

B: before; A: after; TG: triglycerides; c-HDL: high-density lipoprotein cholesterol, Glu: glucose; c-LDL: Low-density cholesterol; TCho: total cholesterol; SBP: systolic blood pressure; DBP: diastolic blood pressure; BMI: body mass composition; WC: waist circumference; SMM: skeletal muscle mass; ST: subcutaneous adipose tissue; BFM: body fat mass; VAT: visceral adipose tissue. Data are presented in media ± SD or median and interquartile interval (IIC). *p* (post hoc): *p* value adjusted with Bonferroni test. # Statistically significant difference between before and after. * Statistically significant difference vs. normal. ^&^ Statistically significant difference vs. overweight (*p* < 0.05).

**Table 3:** Leukocyte distribution before and after of dietary plan according to BMI.

Variable (*n* = 59) (%)	Normal (*n* = 29)	Overweight (*n* = 14)			Obesity (*n* = 16)			*p* (ANOVA)	
		B	A	*p* ^#^	B	A	*p* ^#^	*p*	*p* (post hoc) *^,&^
Total Lymphocytes	30.2 ± 9.7	29.9 (27.8–40.9)	26.1 ± 8.2 ^#^	0.019	30.3 (27.4–32.4)	31.1 ± 8.6	0.798	0.600	
Monocytes	8.3 (6.5–9.9)	7.7 ± 2.3	7.3 (6.6–8)	0.636	6.4 ± 2 *	7.4 (6.9–8.3)	0.061	0.017	0.014
Granulocytes	63.9 (56.5–69.3)	61.5 (53.1–66.3)	66.6 ± 7.6 ^#^	0.016	62.7 (61–66.2)	60.9 ± 8.5	0.530	0.560	
Lymphocytes B (CD19)	12.7 (7.1–23.1)	14.8 (10–23.4)	15.4 (9.9–19.1)	0.635	13.7 (9.4–23.2)	9.3 (7.9–16)	0.052	0.384	
Lymphocytes NK (CD16CD56)	20.4 ± 8.6	14.3 ± 5.1 *	10.9 (7.5–17.6)	0.253	17.2 ± 5.9	13.3 (8.9–23.9)	0.413	0.036	0.041
Lymphocytes T CD3+	63.7 ± 12.7	68.2 ± 8.7	71.4 ± 8.4	0.128	65.6 ± 10.3	72.1 ± 9.3 ^#^	0.012	0.456	
Lymphocytes T CD4+	52.4 ± 11.5	51.9 ± 7.5	53.5 ± 6.8	0.217	56.2 ± 10.6	54.2 ± 10.9	0.455	0.506	
CD4+CD62-	29.1 ± 10	46.4 ± 18.3 *	41.2 ± 13.5	0.538	36 ± 24.7	40.1 ± 15.5	0.644	0.053	0.058
Lymphocytes T CD8+	33.4 ± 9	39.4 ± 13.7	32.6 ± 6.5	0.143	23.9 ± 6.5 ^&^	23.5 ± 9.3	0.871	0.035	0.031
CD8+CD28-	31.7 ± 12.9	34.8 ± 20.5	32 ± 12.9	0.700	25.8 ± 16.2	22.4 ± 9.6	0.476	0.587	
CD3+CD45RA+	53.8 (43.6–61)	36.8 ± 12.9 *	39.4 ± 11.3	0.199	35.5 ± 12.3 *	35.5 ± 12.4	0.988	0.000	0.003
CD3+CD54RO+	32.5 ± 9.5	45.6 ± 10.9 *	44.2 ± 8.2	0.380	40.1 ± 13	42.7 ± 11.3	0.327	0.006	0.008
CD3+ CD45RA+CD45RO+	13.6 ± 4.1	17.7 ± 6.8	14.8 (11–18.1)	0.236	18.8 ± 7.7 *	18.7 (14.4–25.6)	0.494	0.054	0.080
CD4+ CD45RA+	41.5 ± 10.1	24.8 ± 10.6 *	28.1 ± 13	0.183	26.2 ± 8.2 *	26.9 ± 10.2	0.791	0.000	0.000
CD4+ CD45RO+	40.7 ± 10.6	60.1 ± 11.2 *	60.3 ± 12.7	0.944	51.9 ± 12.5 *	58.9 ± 12.3 ^#^	0.016	0.000	0.000
CD4+ CD45RA+CD45RO+	14.1 ± 5.2	14 ± 3.9	11.1 (7.8–14.3)	0.092	17.6 ± 5.8	11.8 (8.6–18.5) ^#^	0.034	0.114	

B: before; A: after. Data are presented in media ± SD or median and interquartile interval (IIC). *p* (post hoc): *p* value adjusted with Bonferroni test. ^#^ Statistically significant difference between before and after. * Statistically significant difference vs. normal. & Statistically significant difference vs. overweight (*p* < 0.05).

**Table 4:** Partial correlations and linear regressions after intervention between leucocytes and biochemical indicators, anthropometric measurements and body composition.

Leukocyte Cells (%)	Variables	*p*	*p*
Monocytes Δ	DBP Δ (mmHg)	−0.555	0.003 *
	ST Δ (%)	0.588	0.013 *
Lymphocytes B Δ	c-LDL Δ (md/dL)	0.370	0.069
Lymphocytes NK Δ	TG Δ (md/dL)	0.431	0.032 *
Lymphocytes T CD4+ Δ	c-HDL Δ (md/dL)	0.339	0.090
	ST Δ (%)	−0.446	0.073
Lymphocytes T CD4+CD62- Δ	WC Δ (cm)	−0.553	0.026 *
	TG Δ (md/dL)	0.365	0.056
Lymphocytes T CD8+CD28- Δ	ST Δ (%)	0.721	0.028 *
T CD4+CD45RA+ Δ	TCho Δ (md/dL)	0.487	0.030 *
	c-LDL Δ (md/dL)	0.474	0.035 *
	WC Δ (cm)	0.575	0.008 *
	c-HDL Δ (md/dL)	0.395	0.085
T CD4+CD45RO+ Δ	Glu Δ (md/dL)	−0.501	0.021 *
	c-HDL Δ (md/dL)	−0.393	0.078
CD4+CD45RO+CD45RA+ Δ	BFM Δ (kg)	−0.516	0.071

DBP: diastolic blood pressure; ST: subcutaneous adipose tissue; c-LDL: low-density cholesterol; TG: triglycerides; c-HDL: high-density lipoprotein cholesterol; WC: waist circumference; Total Cho: total cholesterol; BFM: body fat mass. *p*: *p* value adjusted by gender, age and BMI (*: *p* < 0.05).
